# Reemergence of Lymphocytic Choriomeningitis Mammarenavirus, Germany

**DOI:** 10.3201/eid2903.221822

**Published:** 2023-03

**Authors:** Calvin Mehl, Claudia Wylezich, Christina Geiger, Nicole Schauerte, Kerstin Mätz-Rensing, Anne Nesseler, Dirk Höper, Miriam Linnenbrink, Martin Beer, Gerald Heckel, Rainer G. Ulrich

**Affiliations:** Friedrich-Loeffler-Institut, Greifswald–Insel Riems, Germany (C. Mehl, C. Wylezich, D. Höper, M. Beer, R.G. Ulrich);; German Center for Infection Research, Hamburg–Lübeck–Borstel–Riems, Germany (C. Mehl, R.G. Ulrich);; Zoo Frankfurt, Frankfurt, Germany (C. Geiger, N. Schauerte);; German Primate Center, Leibniz Institute for Primate Research, Göttingen, Germany (K. Mätz-Rensing);; Landeslabor Hessen, Giessen, Germany (A. Nesseler);; Max Planck Institute for Evolutionary Biology, Plön, Germany (M. Linnenbrink);; University of Bern, Institute of Ecology and Evolution, Bern, Switzerland (G. Heckel)

**Keywords:** lymphocytic choriomeningitis mammarenavirus, LCMV, viruses, Germany, reemergence, house mouse

## Abstract

Lymphocytic choriomeningitis mammarenavirus (LCMV) is a globally distributed zoonotic pathogen transmitted by house mice (*Mus musculus*). We report the reemergence of LCMV (lineages I and II) in wild house mice (*Mus musculus domesticus*) and LCMV lineage I in a diseased golden lion tamarin (*Leontopithecus rosalia*) from a zoo in Germany.

Lymphocytic choriomeningitis mammarenavirus (LCMV) is an enveloped virus with a bisegmented genome of single-stranded, ambisense RNA (*1*). The small (S) segment of ≈3,400 nt encodes structural components, including the glycoprotein (GP) and nucleocapsid protein (NP), whereas the large (L) segment of ≈7,200 nt encodes the L (RNA polymerase) and Z proteins (*1*,*2*). First identified in St. Louis, Missouri, USA, in 1933 (*3*), LCMV is a zoonotic pathogen transmitted through contact with excreta and secretions of infected house mice (*Mus musculus*), the reservoir host (*4*). More recently, LCMV RNA was also detected in wild wood mice (*Apodemus sylvaticus*) from Spain (*5*).

In humans, LCMV can cause symptoms ranging from influenza-like illness to meningitis and encephalitis (*6*). Infection during pregnancy may lead to neurologic and developmental problems in infants (*7*). New World primates (family Callitrichidae) are also susceptible to infection, resulting in callitrichid hepatitis, a lethal infection exhibiting histopathologic lesions in the brain, liver, and lymphoid tissues (*8*).

During 1968–1973, a total of 48 human cases of LCMV infection were reported in Germany, many of which originated from pet hamsters (*Mesocricetus auratus*) (*6*,*7*,*9*). Thereafter, 6 prenatal or postnatal infections were reported during 1991–1997 in Germany, most of which were believed to have originated from pet rodents (*10*). During 1999–2000, a total of 4 callitrichid hepatitis cases were reported in Germany: 1 in a Goeldi’s monkey (*Callimico goeldii*) and 3 in pygmy marmosets (*Cebuella pygmaea*) (*8*).

Very little is known on the distribution and prevalence of LCMV in mice from Germany, the most comprehensive study being from Ackermann et al. (*11*) in 1964, which only surveyed the western federal states of Germany (former West Germany). That study found the highest prevalence among house mice in North Rhine–Westphalia, in the western part of West Germany. Most recently, Fornůsková et al. (*12*) screened nearly 800 mice from the Czech Republic and eastern Germany sampled during 2008–2019 but detected LCMV-positive mice only in the Czech Republic.

Four lineages of LCMV are recognized (I–IV). Lineages I and II are the most common sequences worldwide. Lineage III consists of a single strain from Georgia, USA. Only S-segment sequences exist for lineage IV, entirely comprising sequences obtained from wood mice (*Apodemus sylvaticus*) from Spain. Fornůsková et al. (*12*) recently proposed that LCMV lineages I and II are host-specific, whereby lineage I is harbored by the house mouse subspecies *M. m. domesticus* and lineage II by the subspecies *M. m. musculus*. This hypothesis is of particular importance in Europe because *M. m. domesticus* and *M. m. musculus* meet in the house mouse hybrid zone, a ≈2,500-km-long stretch from Scandinavia to the Black Sea. The hybrid zone acts as a barrier to gene flow and the spread of pathogens between the subspecies (*12*,*13*), and, as a consequence, *M. m. domesticus* populations in Germany would be expected to harbor only lineage I.

Although house mice are strongly associated with human settlements, LCMV surveillance in wild mice in Europe is lacking. This study examines the reemergence of LCMV in a golden lion tamarin (*Leontopithecus rosalia*; family Callitrichidae) and wild house mice (*M. m. domesticus*) from a zoo in Germany.

## The Study

In late 2021, an adult golden lion tamarin from a zoo in western Germany died (Appendix). On the basis of the symptoms and an initial diagnosis by the Hessian State Laboratory (Landeslabor Hessen, Giessen, Germany), we conducted further screening for LCMV. The Hessian State Laboratory and the zoo sent tissue samples from the golden lion tamarin and wild mice from the zoo (taken in 2009, 2021, and 2022) to the Friedrich-Loeffler-Institut (Greifswald–Insel Riems, Germany) for molecular and epidemiologic investigations. We extracted and screened nucleic acids for LCMV using conventional reverse transcription PCR (*14*). We detected LCMV RNA in the brain of the golden lion tamarin and in the kidneys of 55% of wild house mice (*M. m. domesticus*) from 2021 and 2022 (n = 53) but not in any of the house mice from 2009 (n = 82). On the basis of ≈340 nt sequences from the L segment of the virus (GenBank accession nos. OP938541–68), we selected 2 mice with the most dissimilar LCMV sequences and, together with brain tissue from the diseased golden lion tamarin, used them for high-throughput sequencing of complete genomes (GenBank accession nos. OP958777–82) (Appendix).

We used the new complete coding-region sequences (L, GP, and NP) together with all published LCMV genomes to reconstruct phylogenetic trees using the general time reversible substitution model with invariable sites and gamma distribution (MrBayes 3.2.7, https://nbisweden.github.io/MrBayes) (*15*). LCMV sequences of the full coding regions of the L, NP, and GP proteins were almost identical between the golden lion tamarin and 1 of the mice, forming a monophyletic clade within LCMV lineage II (Figure; Appendix Figure). The sequences obtained from the other mouse fell within lineage I (Figure; Appendix Figure). According to the L segment sequences (≈340 nt) obtained from the remaining 27 LCMV-positive mice, both lineages were nearly equally represented in the zoo population (lineage I for 16 mice, lineage II for 11 mice).

**Figure:**
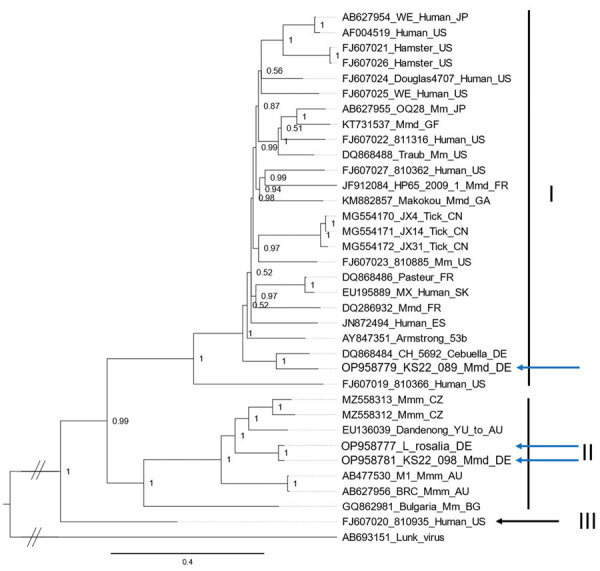
Phylogeny of the L protein encoding nucleotide sequences of lymphocytic choriomeningitis virus (LCMV) identified in Germany (blue arrows) and reference sequences, constructed by using Bayesian inference. Lunk virus from *Mus minutoides* mice was used as an outgroup. Sequence names are comprised of the GenBank accession number, strain name, host species and country of origin (wherever known). Countries are represented by their International Organization for Standardization code (AU, Australia; BG, Bulgaria; CN, China; DE, Germany; ES, Spain; FR, France; GA, Gabon; GF, French Guiana; JP, Japan; SK, Slovakia; US, USA; YU, former Yugoslavia). Roman numerals (I–IV) represent the different LCMV lineages defined according to Albariño et al. (*2*); WE and Armstrong refer to laboratory strains of LCMV. Mm, *Mus musculus*; Mmm, *Mus musculus musculus*; Mmd, *Mus musculus domesticus.*

We obtained sequences from the mitochondrial DNA d-loop of all LCMV-positive mice and several LCMV-negative mice from 2009 (n = 32), 2021 (n = 12), and 2022 (n = 41). All those sequences identified exclusively the house mouse subspecies *M. m. domesticus* (data not shown).

## Conclusions

The high similarity between LCMV lineage II in a golden lion tamarin and a wild house mouse indicates that the virus was passed between wild and captive animals in the zoo. The large number of LCMV lineage I and II strains in the wild house mouse population at this site suggests either an outbreak after recent introduction from 2 different sources or long-term persistence in the local house mouse population but with very low prevalence in 2009.

Despite considerable effort by researchers to detect LCMV in Germany, the virus remains mostly elusive. Although the route through which LCMV entered the zoo is not known, this event most likely occurred after 2009. The virus may have been brought in through naturally occurring wild animals in the region (e.g., wild house mice) or, although unlikely, through infected zoo animals.

We provide evidence for LCMV lineage II in Germany within an area naturally occupied only by the *M. m. domesticus* subspecies of house mice. The occurrence of both LCMV lineages I and II in *M. m. domesticus* mice does not support the subspecies host specificity proposed by Fornůsková et al. (*1*). Further evaluation of LCMV association with house mouse subspecies in Germany and other parts of the world will help clarify potential expanded risk to animal and human health.

Appendix Additional information about reemergence of lymphocytic choriomeningitis mammarenavirus, Germany.
