# Supplementary material for: Reemergence of Lymphocytic Choriomeningitis Mammarenavirus, Germany
Source: Emerg Infect Dis. 2023 Mar;29(3):631–4. doi: 10.3201/eid2903.221822 (PMC9973704; doi:10.3201/eid2903.221822)
Supplement: Appendix — Additional information about reemergence of lymphocytic choriomeningitis mammarenavirus, Germany. [file 22-1822-Techapp-s1.pdf]

Article DOI: https://doi.org/10.3201/eid2903.221822

Reemergence of Lymphocytic Choriomeningitis Mammarenavirus, Germany

Appendix

Methods

Pathology and Epidemiology

In late 2021, an adult golden lion tamarin (*Leontopithecus rosalia*) from a German zoo was found unresponsive and later succumbed to illness. A subsequent necropsy of the monkey at the Landeslabor Hessen revealed localized necrotizing hepatitis, low-grade lymphocytic meningitis, interstitial nephritis and interstitial non-inflammatory pneumonia with localized haemorrhages. Tissues were collected and the initial diagnosis of LCMV was given. Tissue samples were then sent to the Friedrich-Loeffler-Institut (FLI) for further investigation.

To determine whether wild house mice (*Mus musculus*) were harboring and potentially spreading LCMV, mice - collected during routine pest management from sites throughout the zoo (2021–2022) - were frozen at −20°C and sent to the FLI for investigation. Furthermore, mice collected from the same zoo in 2009, stored at −20°C, were also sent to the FLI for investigation. The mice were then thawed at 4°C and dissected under Biosafety level 3 conditions.

Nucleic Acid Analyses

Nucleic acids were isolated from homogenized brain, liver or kidney tissue using NucleoMag® VET (Macherey-Nagel, Düren, Germany), per kit instructions, on a KingFisher Flex Purification System (Thermo Fisher Scientific, Waltham, MA, USA). A one-step RT-PCR was performed using SuperScript III RT-PCR Kit (Qiagen, Hilden, Germany) and arenavirus primers, as described in Vieth et al. (*1*). The products were then resolved by agarose gel electrophoresis (~340 nt segment). BigDye Terminator v1.1 Cycle Sequencing Kit (Applied Biosystems, Waltham, MA, USA) was then used to amplify DNA, which was purified using NucleoSEQ® Columns (Macherey-Nagel, Düren, Germany), and used for Sanger sequencing.

By comparing partial sequences (obtained as above, uploaded to GenBank with accession numbers OP938541 – OP938568) from the mice to that from the golden lion tamarin, a mouse with the most similar and a mouse with the most dissimilar sequences were selected for detailed investigation via high-throughput sequencing (HTS). Brain tissue from the golden lion tamarin and kidney tissue from the two mice were used for HTS. Library preparation and HTS was done as detailed described by Pfaff et al. 2022 (*2*). The taxonomic identification tool RIEMS (*3*) was used for initial detection of potential pathogens contained in the datasets. Full coding sequences of the LCMV L- and S-segments from the golden lion tamarin (accession numbers OP958777 and OP958778, respectively) and both mice (accession numbers OP958779 - OP958782) were then obtained using de novo assembly with the Genome Sequencer software suite (version 2.6; Roche).

Mice were identified to species level using cytochrome *b* mitochondrial DNA sequences, as described in (*4*). Subspecies differentiation was done based on mitochondrial d-loop sequence analysis as described previously by Linnenbrink et al. 2013 (*5*).

Phylogenetic Analyses

Sequences were aligned in BioEdit (*6*) using the ClustalW algorithm, and identified using BLAST (NCBI (*7*)). For the ~340 nt L-segment sequences obtained from the mice, a neighbor-joining tree (Mega11 (*8*)) was constructed to identify clades within all L-segment sequences.

For the full coding region sequences obtained via HTS, JModelTest2 (*9*,*10*) was used to determine the best-fit nucleotide substitution model. The best-fit model for all genes (L, GP and NP) was general time reversible (GTR) with a proportion of invariable sites and gamma distribution. Phylogenetic trees were obtained using Bayesian inference in MrBayes v.3.2.7 (*11*). Twenty million generations were run with trees sampled every 100 generations. The first 25% were discarded as burn-in.

References

1. Vieth S, Drosten C, Lenz O, Vincent M, Omilabu S, Hass M, et al. RT-PCR assay for detection of Lassa virus and related Old World arenaviruses targeting the L gene. Trans R Soc Trop Med Hyg. 2007;101:1253–64. PubMed https://doi.org/10.1016/j.trstmh.2005.03.018

2. Pfaff F, Breithaupt A, Rubbenstroth D, Nippert S, Baumbach C, Gerst S, et al. Revisiting rustrela virus: new cases of encephalitis and a solution to the capsid enigma. Microbiol Spectr. 2022;10:e0010322. PubMed https://doi.org/10.1128/spectrum.00103-22

3. Scheuch M, Höper D, Beer M. RIEMS: a software pipeline for sensitive and comprehensive taxonomic classification of reads from metagenomics datasets. BMC Bioinformatics. 2015;16:69. PubMed https://doi.org/10.1186/s12859-015-0503-6

4. Schlegel M, Ali HS, Stieger N, Groschup MH, Wolf R, Ulrich RG. Molecular identification of small mammal species using novel cytochrome B gene-derived degenerated primers. Biochem Genet. 2012;50:440–7. PubMed https://doi.org/10.1007/s10528-011-9487-8

5. Linnenbrink M, Wang J, Hardouin EA, Künzel S, Metzler D, Baines JF. The role of biogeography in shaping diversity of the intestinal microbiota in house mice. Mol Ecol. 2013;22:1904–16. PubMed https://doi.org/10.1111/mec.12206

6. Hall TA. BioEdit: a user-friendly biological sequence alignment editor and analysis program for Windows 95/98/NT. Nucleic Acids Symp Ser. 1999;41:95–8.

7. Altschul SF, Gish W, Miller W, Myers EW, Lipman DJ. Basic local alignment search tool. J Mol Biol. 1990;215:403–10. PubMed https://doi.org/10.1016/S0022-2836(05)80360-2

8. Tamura K, Stecher G, Kumar S. MEGA11: Molecular Evolutionary Genetics Analysis Version 11. Mol Biol Evol. 2021;38:3022–7. PubMed https://doi.org/10.1093/molbev/msab120

9. Darriba D, Taboada GL, Doallo R, Posada D. jModelTest 2: more models, new heuristics and parallel computing. Nat Methods. 2012;9:772. PubMed https://doi.org/10.1038/nmeth.2109

10. Guindon S, Gascuel O. A simple, fast, and accurate algorithm to estimate large phylogenies by maximum likelihood. Syst Biol. 2003;52:696–704. PubMed https://doi.org/10.1080/10635150390235520

11. Ronquist F, Teslenko M, van der Mark P, Ayres DL, Darling A, Höhna S, et al. MrBayes 3.2: efficient Bayesian phylogenetic inference and model choice across a large model space. Syst Biol. 2012;61:539–42. PubMed https://doi.org/10.1093/sysbio/sys029

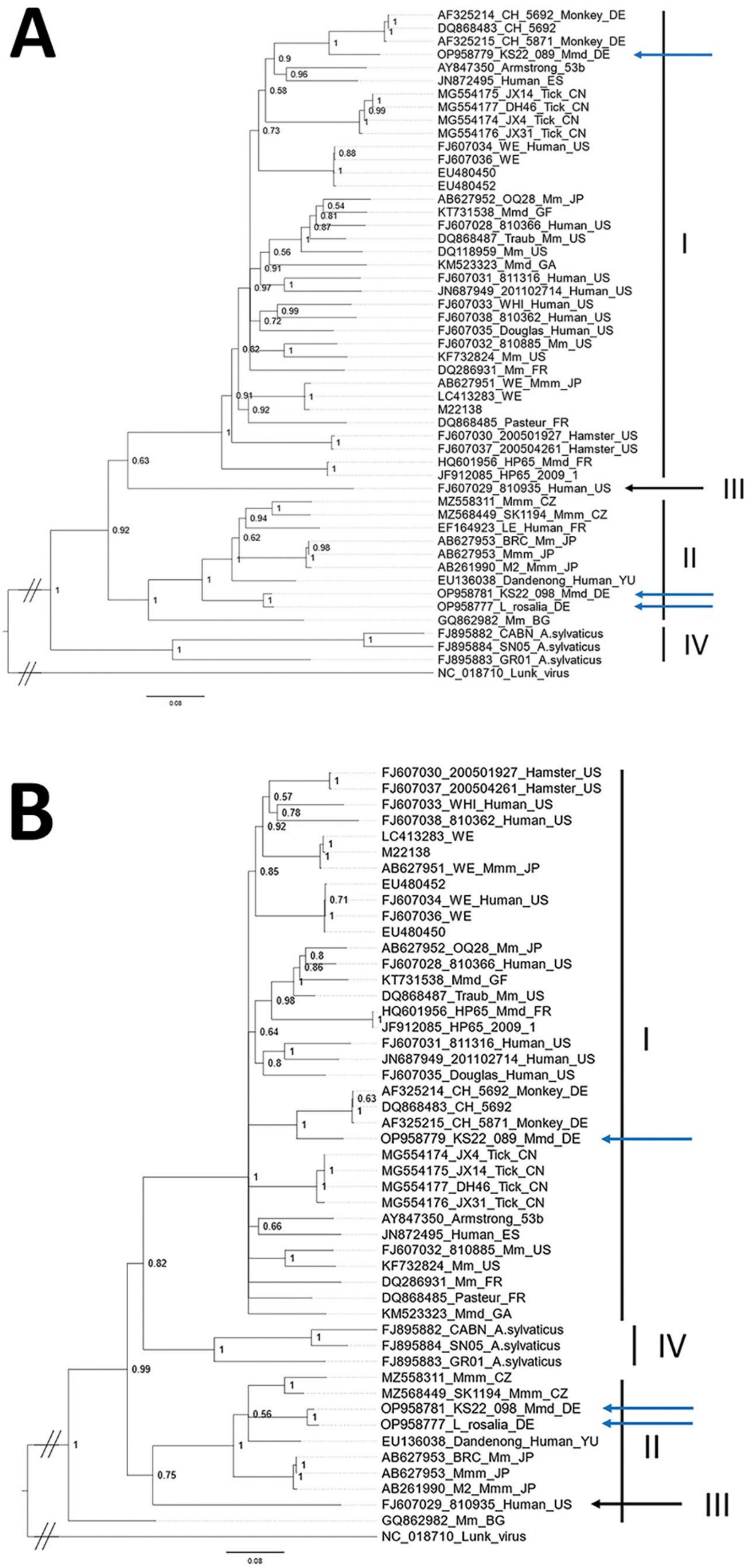
A
AF325214_CH_5692_Monkey_DE
DQ868483_CH_5692
AF325215_CH_5871_Monkey_DE
OP958779_KS22_089_Mmd_DE
AY847350_Armstrong_53b
JN872495_Human_ES
MG554175_JX14_Tick_CN
MG554177_DH46_Tick_CN
MG554174_JX4_Tick_CN
MG554176_JX31_Tick_CN
FJ607034_WE_Human_US
FJ607036_WE
EU480450
EU480452
AB627952_OQ28_Mm_JP
KT731538_Mmd_GF
FJ607028_810366_Human_US
DQ868487_Traub_Mm_US
DQ118959_Mm_US
KM523323_Mmd_GA
FJ607031_811316_Human_US
JN687949_201102714_Human_US
FJ607033_WHI_Human_US
FJ607038_810362_Human_US
FJ607035_Douglas_Human_US
FJ607032_810885_Mm_US
KF732824_Mm_US
DQ286931_Mm_FR
AB627951_WE_Mmm_JP
LC413283_WE
M22138
DQ868485_Pasteur_FR
FJ607030_200501927_Hamster_US
FJ607037_200504261_Hamster_US
HQ601956_HP65_Mmd_FR
JF912085_HP65_2009_1
FJ607029_810935_Human_US
MZ558311_Mmm_CZ
MZ568449_SK1194_Mmm_CZ
EF164923_LE_Human_FR
AB627953_BRC_Mm_JP
AB627953_Mmm_JP
AB261990_M2_Mmm_JP
EU136038_Dandenong_Human_YU
OP958781_KS22_098_Mmd_DE
OP958777_L_rosalia_DE
GQ862982_Mm_BG
FJ895882_CABN_A.sylvaticus
FJ895884_SN05_A.sylvaticus
FJ895883_GR01_A.sylvaticus
NC_018710_Lunk_virus
I
II
III
IV
0.08
B
FJ607030_200501927_Hamster_US
FJ607037_200504261_Hamster_US
FJ607033_WHI_Human_US
FJ607038_810362_Human_US
LC413283_WE
M22138
AB627951_WE_Mmm_JP
EU480452
FJ607034_WE_Human_US
FJ607036_WE
EU480450
AB627952_OQ28_Mm_JP
FJ607028_810366_Human_US
KT731538_Mmd_GF
DQ868487_Traub_Mm_US
HQ601956_HP65_Mmd_FR
JF912085_HP65_2009_1
FJ607031_811316_Human_US
JN687949_201102714_Human_US
FJ607035_Douglas_Human_US
AF325214_CH_5692_Monkey_DE
DQ868483_CH_5692
AF325215_CH_5871_Monkey_DE
OP958779_KS22_089_Mmd_DE
MG554174_JX4_Tick_CN
MG554175_JX14_Tick_CN
MG554177_DH46_Tick_CN
MG554176_JX31_Tick_CN
AY847350_Armstrong_53b
JN872495_Human_ES
FJ607032_810885_Mm_US
KF732824_Mm_US
DQ286931_Mm_FR
DQ868485_Pasteur_FR
KM523323_Mmd_GA
FJ895882_CABN_A.sylvaticus
FJ895884_SN05_A.sylvaticus
FJ895883_GR01_A.sylvaticus
MZ558311_Mmm_CZ
MZ568449_SK1194_Mmm_CZ
OP958781_KS22_098_Mmd_DE
OP958777_L_rosalia_DE
EU136038_Dandenong_Human_YU
AB627953_BRC_Mm_JP
AB627953_Mmm_JP
AB261990_M2_Mmm_JP
FJ607029_810935_Human_US
GQ862982_Mm_BG
NC_018710_Lunk_virus
I
IV
II
III
0.08

Appendix Figure. Phylogeny of the nucleotide sequences of lymphocytic choriomeningitis virus (LCMV) encoding (A) glycoprotein and (B) nucleoprotein identified in Germany (blue arrows) and reference sequences, constructed by using Bayesian inference. Lunk virus from *Mus minutoides* mice was used as an outgroup. Sequence names are comprised of, if known, the GenBank accession number, strain name, host species and country of origin. Countries are represented by their ISO code. Roman numerals (I-IV) represent the different virus lineages as defined according to Albariño et al. 2010 (*1*). "WE" and "Armstrong" are laboratory strains of LCMV. Mm = *Mus musculus*, Mmm = *Mus musculus musculus*, Mmd = *Mus musculus domesticus*, AU = Australia, BG = Bulgaria, CN = China, CZ = Czech Republic, DE = Germany, ES = Spain, FR = France, GA = Gabon, GF = French Guiana, JP = Japan, SK = Slovakia, U.S. = USA, YU = former Yugoslavia.
